# An investigation into augmentation and preprocessing for optimising X-ray classification in limited datasets: a case study on necrotising enterocolitis

**DOI:** 10.1007/s11548-024-03107-0

**Published:** 2024-04-23

**Authors:** Franciszek Nowak, Ka-Wai Yung, Jayaram Sivaraj, Paolo De Coppi, Danail Stoyanov, Stavros Loukogeorgakis, Evangelos B. Mazomenos

**Affiliations:** 1 Wellcome/EPSRC Centre for Interventional and Surgical Sciences, Department of Medical Physics and Biomedical Engineering, UCL, London, UK; 2 Department of Specialist Neonatal and Paediatric Surgery, Great Ormond Street Hospital, NHS Foundation Trust, London, UK

**Keywords:** Data augmentation, Preprocessing, Necrotising enterocolitis, X-ray imaging

## Abstract

**Purpose:**

Obtaining large volumes of medical images, required for deep learning development, can be challenging in rare pathologies. Image augmentation and preprocessing offer viable solutions. This work explores the case of necrotising enterocolitis (NEC), a rare but life-threatening condition affecting premature neonates, with challenging radiological diagnosis. We investigate data augmentation and preprocessing techniques and propose two optimised pipelines for developing reliable computer-aided diagnosis models on a limited NEC dataset.

**Methods:**

We present a NEC dataset of 1090 Abdominal X-rays (AXRs) from 364 patients and investigate the effect of geometric augmentations, colour scheme augmentations and their combination for NEC classification based on the ResNet-50 backbone. We introduce two pipelines based on colour contrast and edge enhancement, to increase the visibility of subtle, difficult-to-identify, critical NEC findings on AXRs and achieve robust accuracy in a challenging three-class NEC classification task.

**Results:**

Our results show that geometric augmentations improve performance, with Translation achieving +6.2%, while Flipping and Occlusion decrease performance. Colour augmentations, like Equalisation, yield modest improvements. The proposed Pr-1 and Pr-2 pipelines enhance model accuracy by +2.4% and +1.7%, respectively. Combining Pr-1/Pr-2 with geometric augmentation, we achieve a maximum performance increase of 7.1%, achieving robust NEC classification.

**Conclusion:**

Based on an extensive validation of preprocessing and augmentation techniques, our work showcases the previously unreported potential of image preprocessing in AXR classification tasks with limited datasets. Our findings can be extended to other medical tasks for designing reliable classifier models with limited X-ray datasets. Ultimately, we also provide a benchmark for automated NEC detection and classification from AXRs.

**Supplementary Information:**

The online version contains supplementary material available at 10.1007/s11548-024-03107-0.

## Introduction

Deep learning (DL) is established as a very promising technology for X-ray analysis and computer-aided diagnosis (CAD), with application in a range of diverse pathologies from fracture to cancer detection [1–3]. The most successful cases of employing DL models for CAD have been reported on relatively large volume datasets containing tens or even hundreds of thousands of images [1–4].

X-ray imaging is routinely used to diagnose rare conditions. One such pathology is necrotising enterocolitis (NEC), a severe intestinal infection affecting premature newborns. Nearly 12% of infants born weighing less than 1500 g will develop NEC, with overall mortality between 18 and 30% and major long-term complications (inflammatory strictures, bowel obstruction, poor neurodevelopment) [5]. Economic and societal impact is high accounting for approximately 19% of neonatal expenditure in the USA [6]. Depending on the severity, medical NEC (mNEC) cases are treated with gut rest, intravenous nutrition (total parenteral nutrition) and antibiotics. Many infants though will require surgical intervention (sNEC) involving intestinal resections and stoma formation, with severe cases constituting surgical emergencies [7]. Mortality rates can reach up to 50% in sNEC cases, and associated morbidity includes severe and chronic complications, such as abdominal contamination due to intestinal leakage, short gut syndrome and enduring neurological impairment [7, 8].

**Fig. 1:**
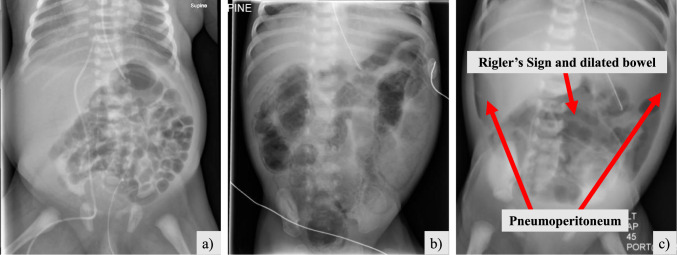
Example of images in our NEC dataset. **a** NP—No Pathology, **b** mNEC—Medical NEC, **c** sNEC—Surgical NEC. Arrows indicate NEC findings

Early diagnosis and staging from abdominal X-rays (AXR), and subsequent surgical referral are vital as delays can negatively impact outcomes. However, the radiological signs of NEC in AXRs are very subtle, making their identification and interpretation challenging, especially for medical professionals without specialised expertise. Confounding factors such as variability in presentation (see Fig. 1) and similarities to other conditions (neonatal sepsis) pose further challenges to radiologists, paediatric surgeons and neonatologists for correct diagnosing, staging and treatment decisioning. Local neonatal ICUs often lack personnel with specialised NEC expertise, which results in delayed diagnosis and patient transfer to a paediatric surgical centre, ultimately delaying initiation of medical treatment or surgical intervention, with potentially severe adverse outcomes [9]. In many cases, extended management with parenteral nutrition is followed which is both costly and may affect liver function [10].

Existing studies on automated NEC diagnosis employed traditional machine learning techniques, leveraging biomarkers and clinical laboratory tests (i.e. tabular data) as key features [11, 12]. Gao et. al. are among the first to utilise AXRs for developing DL models (SENet-154, ResNet-50) for NEC classification. In a private dataset of 4535 images, they report a maximum accuracy of 73.27% in a binary (NEC/no-NEC) classification task. In the same work, features extracted from X-rays were integrated with clinical variables such as heart rate and haemoglobin into a LightGBM model to finalise classification of NEC. The authors also reported utilising a range of image augmentation techniques to increase performance, but their actual effect remains unexplored [13].

Due to the rarity of NEC, the development of CAD methodologies will have to account for limited data availability. A solution to artificially increase the size of datasets is with image augmentation pipelines, where randomised geometrical and image transformations are applied to images with a likelihood during training, also shown to reduce overfitting and improve the generalisability of DL models [14]. For instance, Sirazitdinov et al. investigated the impact of various data augmentations such as contrast adjustment, brightness scaling, gamma correction, flipping, rotation, noise addition and blurring, on the diagnosis of lung pathology using the ChestX-ray14 dataset of 112,120 images. Their results indicate that rotation and flipping are the most effective augmentations, yielding accuracy improvements of 1.9% and 1.5%, respectively, compared to the baseline with no augmentation. Conversely, augmentations involving contrast, gamma correction and blurring led to a marginal performance degradation of 0.1% [4]. Chokchaithanakul et al. explored the effects of data augmentation for out-of-domain tuberculosis screening. Different augmentations including rotation, flipping, brightness scaling, contrast enhancement and gamma correction are applied to a multi-centre dataset comprising 6168 images. Their findings indicate that rotation is the most effective augmentation technique, improving in-domain accuracy by +10.1%. Furthermore, flipping is identified as the most beneficial technique for enhancing out-of-domain accuracy, with an improvement of +4.1% [15].

While image augmentations aim to diversify the training data, image preprocessing focuses on enhancing quality and consistency, thereby highlighting the most important features for the model to learn from. Avşar tested three image preprocessing methods for pneumonia detection on 5856 chest X-rays. Only Wiener filtering is reported with a +6.3% accuracy improvement [16]. Heidari et al., whose work is closest to ours, used erosion and filtering techniques for COVID-19 classification on 8474 chest X-rays, achieving a +6.5% accuracy gain [17]. While previous works have shown promising results with various image augmentation and preprocessing techniques, it is important to note that most of the existing literature on image augmentation focuses on medium-to-large datasets, comprising at least several thousand images. Such techniques may not have a similar effect to constrained medical datasets where only a few hundred to a thousand images are available. Our NEC dataset comprises of only 1090 images, collected over a 10-year period, due to the disease’s rarity.

Limited medical datasets are a prevalent issue that affects CAD development, especially for rare conditions [18]. Preprocessing and augmentation is a key step in DL-based CAD for NEC, and although a plethora of techniques have been reported, predominately for adult chest X-rays datasets, these are not directly applicable to our task due to the intricacies of NEC diagnosis because of confounding and subtle signs. To address this, we conducted this study focusing on augmentation and preprocessing methods tailored for our NEC task. To the best of our knowledge, we are the first to focus on image augmentation on abdominal X-rays with feature-enhancing preprocessing pipelines. Our contributions are summarised as follows: (1) Unlike prior studies, we focus on a rare pathology case attempting a novel CAD task (NEC diagnosis), where obtaining large-scale datasets is difficult or impractical. (2) We propose a previously unreported opportunity of image prepossessing in AXR and showcase its potential for improving classification performance in scenarios constrained by data limitations. (3) We propose two optimised preprocessing pipelines: Pr-1 and Pr-2 to enhance the visibility of critical findings in AXR further improving model performance. (4) Experimental outcomes show a performance increase, with a ResNet-50 model for a three-class NEC classification task, of +6.2% when employing Translation as the augmentation method. Furthermore, the use of our proposed preprocessing pipelines in conjunction with Translation augmentation yields a performance boost of +7.1%, demonstrating marked improvement over the baseline model. Finally, we show that proposed pipelines robustly generalise to unseen data, showing even higher gains against the baseline (+13%). Our approach of enhancing model development via the optimum combination of augmentation and preprocessing is directly transferable to other X-ray CAD tasks, especially for rare diseases.

**Table 1:** List of augmentation/preprocessing techniques and their corresponding settings.

Geometric augmentations		Colour augmentations		Preprocessing + combinations	
Name	Settings	Name	Settings	Name	Settings
Baseline	N/A	Noise	(10, 25, 50)% C	Translation & rotation	10% L & 20^∘^ D
Translation	(10, 25, 50)% L	Equalisation	(10, 25, 50)% C	Sharpening & equalisation	10% C & 50% C
Cropping	(159, 174, 188, 200) WH	Sharpening	(10, 25, 50)% C	Pr-1 preprocessing	N/A
Rotation	10, 20, 40, 60	Colour inversion	(10, 25, 50)% C	Pr-2 preprocessing	N/A
Horizontal flipping	(10, 25, 50)% C			Pr-1 & translation	N/A & 10% L
Vertical flipping	(10, 25, 50)% C			Pr-2 & translation	N/A & 10% L
Horizontal & vertical flipping	(10, 25, 50)% C				
Occlusion	(5, 15, 25)% A				

**C**—Chance of augmentation being applied, **L**—Length of image, **WH**—Cropped image width and height, **D**—Degree of rotation, **A**—Area of image

## Methods

### Dataset and model

A fully anonymised dataset was collected from the Great Ormond Street Hospital, London, UK (GOSH). AXRs images from various hardware systems, including mobile X-rays machines, were reviewed by 5 radiologists and paediatric surgeons and labelled in three classes: surgical NEC (sNEC, 372 images from 137 patients), medical NEC (mNEC, 341 images from 102 patients) and No Pathology (NP, 377 images from 143 patients). Example images and the dataset’s demographic information are provided in Fig. 1 and Table 1 in Online Resource 1, respectively.

We employ the established ResNet-50 [19, 20] model initialised on ImageNet [21] and train it, using multiclass cross-entropy loss, the Adam optimiser with a learning rate of 0.001 and batch size of 16, for 150 epochs. We report results on the best-performing setting, comparing them against the baseline with no preprocessing or augmentations using averages, standard deviations and p-values of accuracy, precision, recall and F1 score. The output of the network is a probability value for each class, and the one with the highest is selected as the final class label. Implementation took place on PyTorch and trained on a single RTX 2080-Ti GPU.

**Fig. 2:**
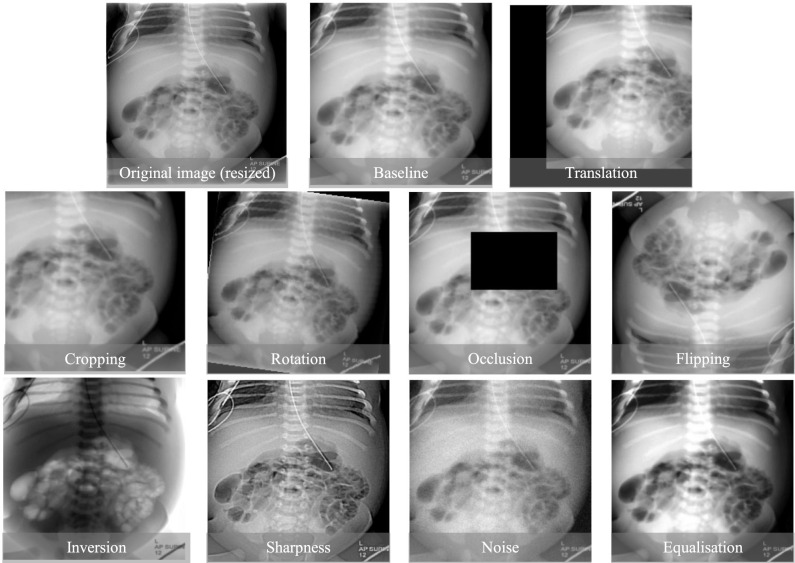
Example of different augmentations applied in our experiments

### Preprocessing and augmentation techniques

We conduct a comprehensive evaluation of image augmentations in alignment with prior studies [4, 15]. All experiments and their settings are listed in Table 1. For geometric augmentations, we examine five geometric augmentations, namely: Translation—to enhance the model’s invariance to the positioning of features; Cropping—to diversify feature sizes and locations while maintaining critical information; Rotation—to enrich the model’s understanding of features across different orientations; Horizontal and Vertical Flipping—to improve the model’s resilience to various spatial configurations. Comprehensively, we examine five colour augmentations: Occlusion—to train the model in recognizing partially visible features, Noise—to increase the model’s robustness against small perturbations; Equalisation—to enhance the contrast and highlight feature distinctions; Sharpening—to emphasise edge details for better feature extraction; and colour inversion—to promote colour invariance in the model’s feature recognition capabilities. Furthermore, we explore the compounding effects of combined augmentations by pairing Translation with Rotation and Sharpening with Equalisation, identified as the top-performing augmentation in their respective categories. Figure 2 shows an example of each augmentation considered.

**Table 2:** NEC classification results from cross-validation and testing experiments

*Fivefold cross-validation*	Accuracy%	Precision%	Recall%	F1
0. Baseline	63.9 ± 2.0	63.7 ± 2.5	63.4 ± 2.4	63.0 ± 2.2
1. Translation	*70.1* ± 1.4*	69.0 ± 1.8*	68.4 ± 2.1*	***68***.***8*** ± ***2***.1*
2. Cropping	69.1 ± 2.2*	68.6 ± 2.6*	68.0 ± 2.3*	67.7 ± 2.2*
3. Rotation	70.0 ± 1.8*	*68.8*±1.8*	***68***.***9*** ± ***1***.7*	68.7 ± 1.7*
4. Horizontal flipping	64.3 ± 1.6	62.7 ± 1.6	62.7 ± 1.7	62.5 ± 1.5
5. Vertical flipping	62.1 ± 4.0	61.2 ± 4.3	60.5 ± 3.9	60.2 ± 3.7
6. Horizontal & vertical flipping	63.8 ± 1.4	63.2 ± 1.2	61.4 ± 1.8	61.3 ± 1.8
7. Occlusion	59.6 ± 2.0	58.6 ± 1.6	57.9 ± 2.3	57.4 ± 2.8
8. Noise	62.6 ± 2.7	61.9 ± 2.2	61.7 ± 2.5	61.4 ± 2.6
9. Sharpening	62.6 ± 1.0	63.2 ± 3.2	61.3 ± 2.2	60.8 ± 2.1
10. Equalisation	*64.6* ± *1.1*	*63.4* ± *1.0*	*63.6* ± *1.0*	*63.2* ± *1*.*0*
11. Colour inversion	60.9 ± 1.8	59.6 ± 1.7	59.5 ± 1.8	59.4 ± 1.8
12. Pr-1 preprocessing	*66.3* ± 1.2*	*64.9* ± *1.3*	*64.6* ± *1.3*	*64.5* ± *1.4*
13. Pr-2 Preprocessing	65.6 ± 2.4	64.4 ± 2.3	64.3 ± 2.3	64.2 ± 2.3
14. Translation & rotation	67.2 ± 1.3*	66.4 ± 1.8*	64.6 ± 1.9	64.6 ± 2.2
15. Sharpening & equalisation	61.9 ± 2.3	61.1 ± 2.4	60.9 ± 2.3	60.7 ± 2.2
16. Pr-1 & translation	***71***.***0*** ± ***1***.6*	***69***.***7*** ± ***1***.7*	*68*.*7* ± *2*.8*	*68*.*4* ± *3*.1*
17. Pr-2 & translation	69.7 ± 1.2*	68.8 ± 1.4*	67.5 ± 1.9*	67.4 ± 1.9*

*Hold-out testing*	Accuracy%	Precision%	Recall%	F1
Baseline	56.3 ± 1.7*	59.1 ± 1.8*	56.3 ± 2.1*	56.1 ± 2.1*
Translation	68.0 ± 1.4*	**70**.**6** ± 1.5*	68.0 ± 1.3*	67.9 ± 1.4*
Rotation	68.7 ± 1.5*	70.3 ± 1.7*	68.7 ± 1.7*	68.6 ± 1.7*
Pr-1 preprocessing	64.0 ± 1.9*	64.8 ± 2.1*	64.0 ± 2.1*	63.3 ± 2.0*
Pr-2 preprocessing	61.0 ± 2.6*	62.2 ± 1.3*	61.0 ± 2.2*	60.9 ± 2.3*
Pr-1 & translation	**69**.**3** ± 1.7*	70.5 ± 1.8*	**69**.**3** ± 1.6*	**69**.**3** ± 1.7*
Pr-2 & translation	66.3+2.0*	68.0 ± 1.9*	66.3 ± 1.8*	66.0±1.8*

**Fig. 3:**
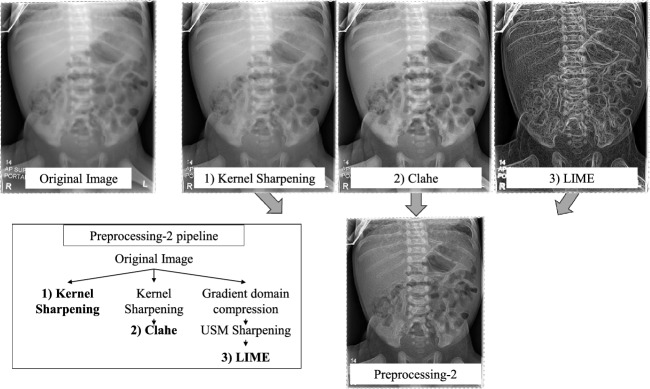
Overview of the Pr-2 pipeline. The pipeline converts the single-channel image into a three channel and sharpens the first channel using a sharpening kernel. The second channel incorporates both sharpening kernel and CLAHE for enhanced contrast. The third leverages histogram compression, unsharp masking, and Low Illumination Image Enhancement (LIME) to emphasise edge-defining structures

With the augmentation and preprocessing methods considered. The best model within each category is in italic, and best overall result is indicated in bold. * indicates *p* < 0.05. For conciseness, only the top-performing settings for each augmentation type are displayed. Full results are provided in Online Resource 1 (Fig. 3)

In addition to the image augmentations listed in Fig. 2, we introduce two image preprocessing pipelines, to improve the model’s focus on key features, highlighting depth in the structures and minimising the impact of irrelevant data on convergence. Pipeline 1 (Pr-1) employs the two most effective colour scheme augmentations (Sharpening and Equalisation, based on experiment results in Table 2), applying them with a 100% likelihood. Drawing inspiration from [22], we also present pipeline 2 (Pr-2), depicted in Fig. 3. Pr-2 is designed to highlight internal structures by stacking three differently processed versions of the original image into a three-channel format. The first channel undergoes sharpening through a sharpening kernel. The second channel receives both a sharpening kernel and Contrast-Limited Adaptive Histogram Equalisation (CLAHE) for enhanced contrast. Lastly, the third channel is processed using histogram compression, unsharp masking and Low Illumination Image Enhancement (LIME) [23] to emphasise edge-defining structures in the abdomen. Experiments are conducted on these two proposed preprocessing pipelines, both individually and in combination with the top-performing augmentation technique, identified as translation.

## Results

For experimentation, following [24–26], we perform fivefold cross-validation to ensure the robustness of our result and avoid overfitting due to small dataset size. We divide the dataset into fivefolds following an 80/20 split for training and validation. Each fold contains the same number (320) of images for each label and is divided into five subsets of 64 images each, with four intended for training and one for validation. The additional 32 sNEC, 1 mNEC and 37 NP AXRs, randomly selected, were used exclusively for validation (see Online Resource 1 for an illustration of the dataset splits). In all five iterations a new model is independently trained with fourfold and validated on onefold that is different during each iteration. It is therefore guaranteed that there is no data leakage between the training and validation sets. In addition to the validation, we further test best-performing setting on a hold-out set of 60 images (20 mNEC, 20 sNEC, 20 no pathology) from 21 patients, to confirm generalisability and robustness of our results.

**Fig. 4:**
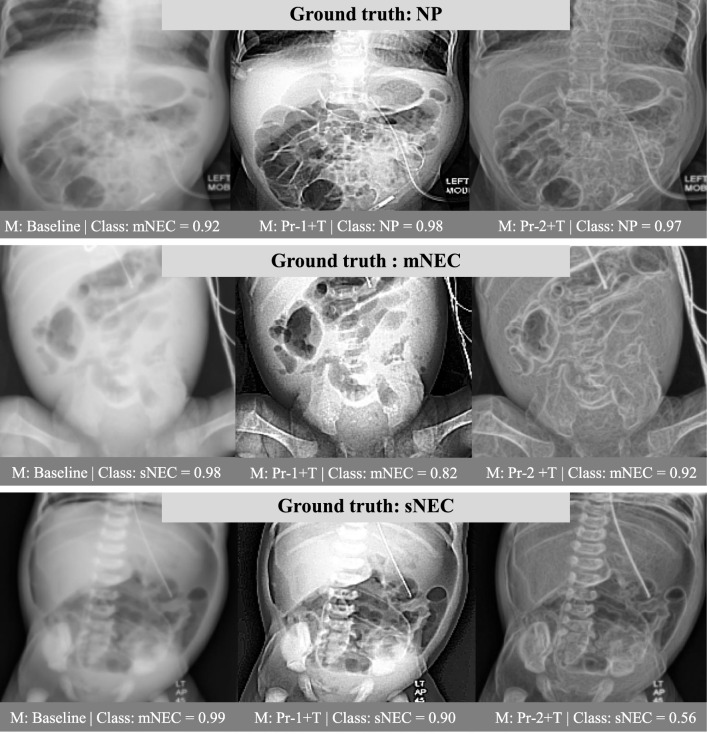
Comparison of predictions between (left) baseline, (middle) Pr-1+Trans, and (right) Pr-2+Trans for all three NEC classes. Preprocessing approaches enhance the signs of NEC within the image, allowing the model to accurately classify the condition

**Fig. 5:**
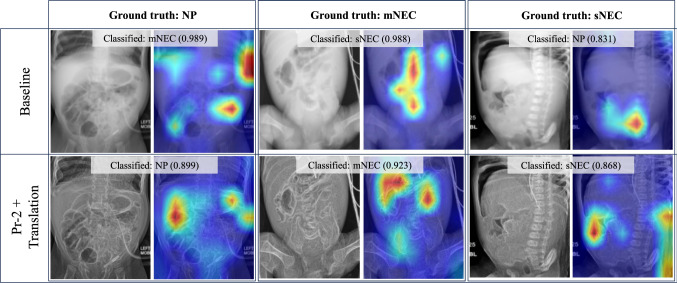
Grad-CAM++ regions contributing to predictions of NEC classes. The rows indicate baseline and Pr-2 & Translation trained models, while columns show input data, truth label and gradient maps along with classification and confidence level

### Cross-validation experiment

Table 2 lists a summary of the results from our NEC classification experiments (full table in the Online Resource 1). Geometric augmentations (1–7) show considerable improvement over the Baseline (0), with Translation (1) achieving the highest of +6.2%. Horizontal and Vertical Flipping and their combination (4–6) have minimum effect. This contradicts [14], suggesting that the ResNet model learns mostly localised representations, when trained in our limited NEC dataset, and finds it difficult to generalise to patterns in widely different locations. Colour scheme augmentations (9–11) lead to small changes, while adding Occlusion and Noise (7,8) reduces performance. Both preprocessing methods (12,13) lead to improvements of +2.4% and +1.7% compared to the Baseline. Combining Translation and Rotation (14) leads to an improvement of + 3.3%, but a smaller one than the individual gains, while combining Sharpening and Equalisation (15) causes a negative effect. Pairing both of the proposed prepossessing pipelines with Translation (16–17) leads to significant improvements with Pr-1 and Translation (17) achieving the best performance overall with an accuracy increase of +7.13% compared to the Baseline.

### Hold-out testing experiment

The bottom section of Table 2 shows results of the best-performing methods on the hold-out testing set. Our previous findings are also confirmed in this experiment. First, all models outperform the baseline and show good generalisation to the unseen dataset. Secondly Rotation (68.70% acc) and Translation (68.0% acc) generalise better than Pr-1 (64.00% acc) and Pr-2 (61.0% acc). This makes sense, as geometric augmentations are expected to provide more resilience to changes in feature representation than preprocessing. Finally, the best performance was again achieved by the model trained using the combination of augmentation and preprocessing (Pr1+trans: 69.3% acc), showing the ability of the proposed approach to effectively generalise to unseen images.

### Discussion and visualisation

Figure 4 compares example predictions across the baseline, Pr-1 with Translation and Pr-2 with Translation. In the first row an NP image is incorrectly classified by the baseline as mNEC and with very high probability. Moreover, in the second and third rows, the baseline model erroneously classifies mNEC as sNEC and sNEC as mNEC, respectively. In the absence of preprocessing, input images appear blurry and marred by low contrast, leading to incorrect model predictions. The application of Pr-1 and Pr-2 effectively sharpens both contrast and edges. This results in enhanced visibility of NEC indicators and findings, allowing the model to learn to distinguish the three classes, yielding accurate predictions.

To further illustrate the improvements added by the Pr-2 & Translation, Fig. 5 shows Grad-CAM++ outputs of validation samples. Evidently, the proposed pipeline better focuses on abdominal areas, where NEC findings are located, leading to correct classification. This is different from the baseline that occasionally focuses on irrelevant areas (e.g. the spine column).

Our results show that the dataset size is an important factor when considering image augmentation and preprocessing pipelines in DL development for X-ray CAD tasks, as it can significantly influence the performance of the network. Contrary to the available literature, for small datasets, augmentations that conserve most of the original information (1–2) tend to be more efficient, while transformations that either significantly change the location of the features (5,6) or occlusions which alter core image information (7), tend to work poorly. With this in mind, when designing an augmentation pipeline, caution is advised when applying multiple augmentation steps, as their combined effect can reduce the model’s performance as indicated in our results (14,15). Large datasets cause a slower model convergence but benefit from pipelines targeting to filter out outliers, thus increasing the robustness of classification. In limited-size datasets, like the one we present here, the main goal should be to artificially expand the available data for training without altering key information. Our results also highlight the potential benefit of enhancing core image attributes (e.g. contrast) via preprocessing to improve classification performance. Both proposed Pr-1 (17.) and Pr-2 (16.) preprocessing pipelines lead to improvements of +2.4% and +1.7% in classification accuracy and increased robustness, as indicated by reduced deviation in the majority of metrics.

It is important to acknowledge that our study has potential limitations. All images are collected from the same medical centre; therefore, our dataset may not represent the full distribution of NEC cases. Also, our two proposed preprocessing pipelines focus on better highlighting the abdominal structures in AXRs, thus enhancing NEC features, but particularly Pr-2 was designed considering the available dataset. This could introduce bias and may make the model perform differently in images from other sources. However, considering our proposed pipeline Pr1+Translation shows that even standard preprocessing techniques (sharpening and equalisation) can increase model’s robustness. Given its simplicity it is expected to introduce minimum bias and be widely generalisable across the spectrum of AXR images.

## Conclusion

This study explores the efficacy of various image augmentations and preprocessing strategies in the development of CAD models for NEC, a rare but devastating condition affecting premature-born babies. The task itself presents major clinical—identification of disease severity, as opposed to simple detection, is the key objective; imaging—subtle and difficult to distinguish radiological signatures; and technical—limited data size due to rarity, challenges. Our exhaustive experimental evaluation reveals that geometric augmentations preserving critical image features are most conducive for model training, showing an increase in performance of +6.2% with Translation and +6.1% with Rotation. Conversely, augmentations such as occlusion, which significantly alter core image information, led to a performance decrement. Colour augmentations, on the other hand, yielded marginal gains, with a maximum improvement of +0.7% achieved through Equalisation. We propose two optimised preprocessing pipelines in a novel X-ray classification task in limited settings: Pr-1, centred on colour and contrast enhancement, and Pr-2, centred on edge enhancement. These pipelines successfully elevated model accuracy by +2.4% and +1.7%, respectively. Moreover, we demonstrated that combining Pr-1 and Pr-2 with Translation augmentation yielded the best outcomes improving classification accuracy by +7.1% against the baseline. In experiments with a hold-out testing set, the proposed pipelines show good ability to generalise to unseen data, while the best-performing one (Pr1 & Translation) achieves the maximum improvement of +13% against the baseline. This investigation provides a nuanced understanding of DL development in X-ray classification, specifically highlighting the critical role of data augmentation and preprocessing techniques in enhancing performance, particularly in the challenging context of limited datasets for automated diagnosis of rare medical conditions. In the future we plan to integrate AXR datasets from different medical centres and investigate any potential domain shift caused by patient distribution, X-ray machine settings, as well as benchmark our proposed pipelines on additional DL architectures (CNN and Transformer-based backbones).

### Supplementary Information

Below is the link to the electronic supplementary material.

Supplementary file 1 (pdf 584 KB)

## Data Availability

Corresponding code and the full dataset will be made available, upon paper acceptance, for research purposes.
