# Supplementary material for: An investigation into augmentation and preprocessing for optimising X-ray classification in limited datasets: a case study on necrotising enterocolitis
Source: Int J Comput Assist Radiol Surg. 2024 Apr 23;19(6):1223–31. doi: 10.1007/s11548-024-03107-0 (PMC11178627; doi:10.1007/s11548-024-03107-0)
Supplement: Supplementary file 1 — (pdf 584 KB) [file 11548_2024_3107_MOESM1_ESM.pdf]

Supplementary materials ("Online Resource 1") for: An Investigation into Augmentation and Preprocessing for Optimizing X-ray Classification in a Limited Dataset: A Case Study on Necrotising Enterocolitis

---

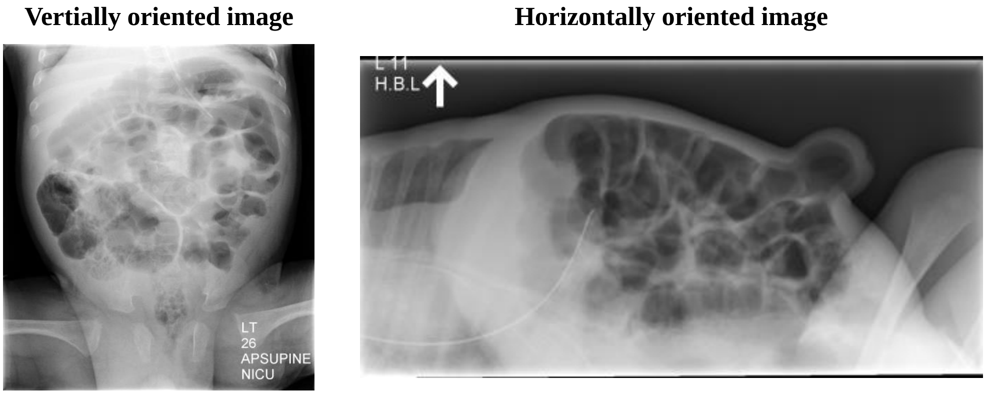

(a) Example of vertically and horizontally oriented images in our dataset.

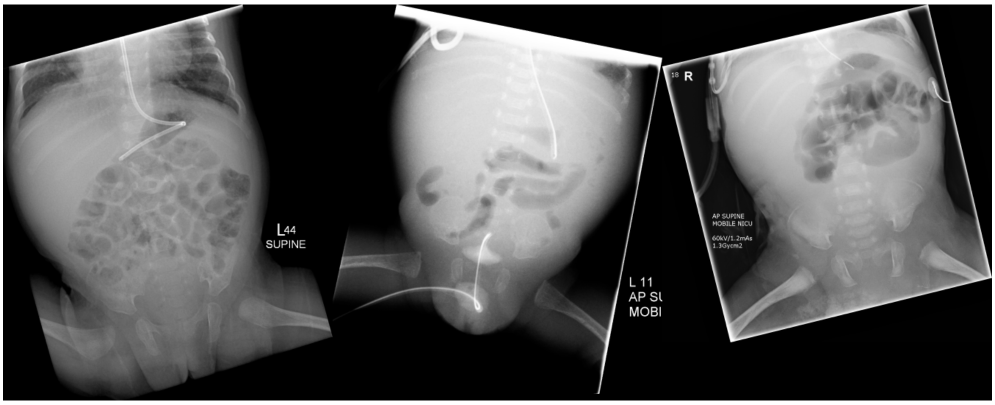

(b) Example of variations of orientation of images in our dataset

Fig. 1: Example of images orientations in our dataset.

Table 1: Numbers and demographic distribution of our Necrotising Enterocolitis dataset

	SN	MN	NP
**Number of images**	372	341	377
**Number of patients**	137	102	143
**Male (%)**	85 (62%)	64 (63%)	79 (55%)
**Median (IQR) Gestation at Birth [weeks]**	28 (13)	35 (10)	38 (3)
**Median (IQR) Birth Weight [kg]**	1.02 (1.13)	2.11 (1.60)	2.98 (1.20)
**Median (IQR) Age at diagnosis [days]**	13 (25)	15 (25)	4 (32)
**Cardiac Comorbidity (%)**	27 (20%)	39 (38%)	76 (53%)

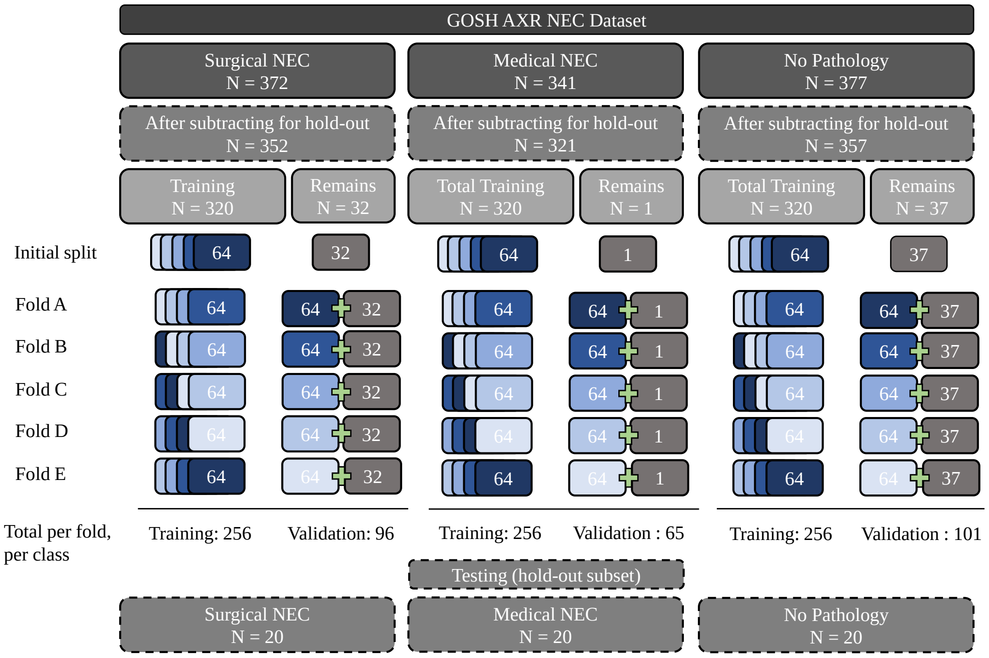

Fig. 2: Schematic showcasing distrubution of images for 5-fold cross validation and hold-out testing. Initially, 20 images from each class are pulled at random to create a hold-out subset. Later, for training and validation remaining images from each class are split into five, and each fold gets assigned 320 images of a given class. Four folds are for training and one for validation at any particular time. The remaining set of 70 randomly pulled AXRs (32 sNEC, 1 mNEC, 37 NP) is used solely for validation. After training, the best performing models are evaluated on the hold-out dataset.

Experiment	Var	Acc %	Acc Pval	Pre %	Pre Pval	Rec %	Rec Pval	F1 %	F1 Pval
Baseline	N/A	63.88±1.96	0.507	63.66±2.51	0.509	63.36±2.43	0.511	62.98±2.24	0.506
**Translation**	**10% r**	**70.11±1.44**	**0.001<**	**69.01±1.78**	**0.002**	**68.43±2.14**	**0.006**	**68.77±2.12**	**0.002**
Translation	25% r	66.24±1.51	0.035	66.34±1.62	0.041	62.98±3.41	0.586	62.33±4.32	0.617
Translation	50% r	62.05±1.18	0.943	62.04±2.47	0.839	59.85±2.54	0.974	59.27±2.72	0.978
Cropping	159x159	64.56±2.48	0.327	63.43±2.25	0.568	61.78±1.64	0.877	61.43±1.95	0.867
Cropping	174x174	67.60±2.14	0.011	66.04±1.78	0.063	64.71±2.59	0.216	64.44±2.91	0.202
Cropping	188x188	68.21±0.43	0.001	67.17±1.50	0.014	66.00±2.04	0.051	65.76±2.20	0.041
**Cropping**	**200x200**	**69.13±2.22**	**0.002**	**68.6±2.64**	**0.008**	**68.02±2.33**	**0.007**	**67.73±2.19**	**0.005**
Rotation	10°	68.67±2.34	0.004	67.32±2.54	0.026	66.92±2.92	0.036	66.87±2.94	0.023
**Rotation**	**20°**	**70.02±1.78**	**0.001<**	**68.79±1.83**	**0.002**	**68.99±1.66**	**0.001**	**68.74±1.69**	**0.001**
Rotation	40°	68.37±1.15	0.001	67.60±1.32	0.008	66.87±1.67	0.015	66.67±1.17	0.005
Rotation	60°	66.62±2.35	0.042	65.70±2.48	0.119	64.82±3.01	0.217	64.54±3.12	0.197
Horizontal Flipping	10% c	63.73±2.50	0.547	61.98±2.11	0.863	60.94±2.49	0.925	60.74±2.67	0.909
**Horizontal Flipping**	**25% c**	**64.33±1.64**	**0.359**	**62.65±1.55**	**0.776**	**62.74±1.74**	**0.685**	**62.47±1.54**	**0.665**
Horizontal Flipping	50% c	64.11±1.39	0.427	63.46±1.57	0.569	62.96±1.49	0.632	62.74±1.31	0.589
**Vertical Flipping**	**10% c**	**62.13±3.99**	**0.799**	**61.15±4.31**	**0.857**	**60.47±3.85**	**0.907**	**60.23±3.66**	**0.907**
Vertical Flipping	25% c	60.68±2.67	0.968	58.78±2.31	0.994	57.71±1.94	0.998	57.55±1.75	0.999
Vertical Flipping	50% c	62.05±1.93	0.912	61.48±1.65	0.932	60.45±2.81	0.944	60.14±2.78	0.945
Hor/Ver Flipping	10% c	63.73±1.28	0.563	63.18±1.77	0.642	61.02±2.18	0.930	60.71±2.43	0.922
Hor/Ver Flipping	25% c	63.65±0.83	0.598	62.52±1.13	0.817	61.57±1.74	0.897	61.38±1.73	0.884
**Hor/Ver Flipping**	**50% c**	**63.80±1.36**	**0.535**	**63.20±1.17**	**0.653**	**61.43±1.81**	**0.909**	**61.29±1.78**	**0.893**
**Erasion (Occlusion)**	**05% r**	**59.62±2.04**	**0.995**	**58.61±1.65**	**0.997**	**57.85±2.26**	**0.997**	**57.38±2.77**	**0.996**
Erasion (Occlusion)	15% r	56.88±2.85	0.999	57.38±1.24	>0.999	55.49±3.55	0.998	54.8±4.20	0.998
Erasion (Occlusion)	25% r	53.08±2.93	>0.999	51.44±2.76	>0.999	50.42±2.93	>0.999	49.99±2.97	>0.999
**Noise**	**10% c**	**62.59±2.71**	**0.796**	**61.91±2.22**	**0.868**	**61.74±2.46**	**0.844**	**61.42±2.61**	**0.834**
Noies	25% c	61.75±1.46	0.956	60.22±1.71	0.983	60.09±1.82	0.980	59.96±1.76	0.979
Noies	50% c	60.23±1.95	0.991	59.70±1.78	0.990	59.65±2.06	0.985	59.23±2.04	0.989
Equalisation	10% c	63.65±2.67	0.564	62.28±2.44	0.805	61.72±2.75	0.834	61.28±3.03	0.833
Equalisation	25% c	63.35±1.39	0.688	62.78±2.20	0.723	62.44±1.97	0.745	62.10±1.72	0.754
**Equalisation**	**50% c**	**64.56±1.12**	**0.268**	**63.38±0.95**	**0.602**	**63.36±1.02**	**0.512**	**63.18±0.96**	**0.434**
**Sharpening**	**10% c**	**62.59±0.99**	**0.888**	**63.19±3.17**	**0.608**	**61.27±2.23**	**0.908**	**60.78±2.11**	**0.929**
Sharpening	25% c	61.75±3.30	0.876	61.68±3.01	0.859	60.89±2.38	0.933	60.51±2.31	0.940
Sharpening	50% c	61.67±2.17	0.935	61.03±2.43	0.938	60.59±2.82	0.936	60.23±2.83	0.939
**Colour Inversion**	**10% c**	**60.91±1.79**	**0.981**	**59.61±1.73**	**0.992**	**59.45±1.78**	**0.991**	**59.39±1.81**	**0.989**
Colour Inversion	25% c	58.10±1.21	>0.999	56.95±1.23	>0.999	56.80±1.24	>0.999	56.63±1.26	>0.999
Colour Inversion	50% c	57.49±0.98	>0.999	57.1±1.310	>0.999	56.18±2.06	>0.999	55.63±2.06	>0.999
**Pr-1**	**N/A**	**66.31±1.16**	**0.024**	**64.89±1.25**	**0.185**	**64.60±1.26**	**0.176**	**64.54±1.37**	**0.110**
Pr-2	N/A	65.55±2.37	0.134	64.42±2.26	0.322	64.34±2.27	0.270	64.20±2.29	0.211
10% Sharp + 50% Equa	N/A	61.90±2.29	0.910	61.05±2.35	0.939	60.87±2.26	0.938	60.73±2.22	0.929
10% Trans + 20° Rot	N/A	67.22±1.33	0.007	66.37±1.78	0.044	64.64±1.85	0.194	64.64±2.16	0.134
**Pr-1 & Transl**	**10% r**	**71.03±1.62**	**0.001<**	**69.70±1.73**	**0.001**	**68.69±2.84**	**0.006**	**68.42±3.11**	**0.007**
Pr-2 & Transl	10% r	69.66±1.24	0.001<	68.80±1.42	0.002	67.47±1.90	0.009	67.41±1.94	0.005

Fig. 3: Table showing full results. The best experiments out of each augmentation/-category are boldened. *Var* - Variable; *Acc* - Accuracy; *Pval* - P-value; *Pre* - Precision; *Rec* - Recall; *Hor/Ver* - Horizontal/Vertical; *Pr-1* - Preprocessing Pipeline 1; *Pr-2* - Preprocessing Pipeline 2; *Sharp* - Sharpness; *Equal* - Equalisation; *Transl* - Translation; *r* - range; *c* - chance.
